# Effect of Transgenic Rootstock Grafting on the Omics Profiles in Tomato

**DOI:** 10.14252/foodsafetyfscj.D-20-00032

**Published:** 2021-06-25

**Authors:** Hiroaki Kodama, Taira Miyahara, Taichi Oguchi, Takashi Tsujimoto, Yoshihiro Ozeki, Takumi Ogawa, Yube Yamaguchi, Daisaku Ohta

**Affiliations:** 1 Graduate Scholl of Horticulture, Faculty of Horticulture, Chiba University, 1-33 Yayoi, Inage-ku, Chiba 263-8522, Japan.; 2 Faculty of Life and Environmental Sciences, University of Tsukuba, 1-1-1 Tennodai, Tsukuba 305-8572, Japan.; 3 Tsukuba Plant Innovation Research Center, University of Tsukuba, 1-1-1 Tennodai, Tsukuba 305-8572, Japan.; 4 Department of Biotechnology and Life Science, Faculty of Engineering, Tokyo University of Agriculture and Technology, 2-24-16 Naka-cho, Koganei 184-8588, Japan.; 5 Graduate School of Life and Environmental Sciences, Osaka Prefecture University, 1-1 Gakuen-cho, Naka-ku, Sakai 599-8531, Japan.

**Keywords:** genetically modified (GM) plants, grafting, new plant breeding technology (NPBT), omics analysis, *Solanum lycopersicum*, tomato

## Abstract

Grafting of non-transgenic scion onto genetically modified (GM) rootstocks provides superior agronomic traits in the GM rootstock, and excellent fruits can be produced for consumption. In such grafted plants, the scion does not contain any foreign genes, but the fruit itself is likely to be influenced directly or indirectly by the foreign genes in the rootstock. Before market release of such fruit products, the effects of grafting onto GM rootstocks should be determined from the perspective of safety use. Here, we evaluated the effects of a transgene encoding β-glucuronidase (GUS) on the grafted tomato fruits as a model case. An edible tomato cultivar, Stella Mini Tomato, was grafted onto GM Micro-Tom tomato plants that had been transformed with the *GUS* gene. The grafted plants showed no difference in their fruit development rate and fresh weight regardless of the presence or absence of the *GUS* gene in the rootstock. The fruit samples were subjected to transcriptome (NGS-illumina), proteome (shotgun LC-MS/MS), metabolome (LC-ESI-MS and GC-EI-MS), and general food ingredient analyses. In addition, differentially detected items were identified between the grafted plants onto rootstocks with or without transgenes (more than two-fold). The transcriptome analysis detected approximately 18,500 expressed genes on average, and only 6 genes were identified as differentially expressed. Principal component analysis of 2,442 peaks for peptides in proteome profiles showed no significant differences. In the LC-ESI-MS and GC-EI-MS analyses, a total of 93 peak groups and 114 peak groups were identified, respectively, and only 2 peak groups showed more than two-fold differences. The general food ingredient analysis showed no significant differences in the fruits of Stella scions between GM and non-GM Micro-Tom rootstocks. These multiple omics data showed that grafting on the rootstock harboring the *GUS* transgene did not induce any genetic or metabolic variation in the scion.

## 1. Introduction

Advances in plant molecular biology to introduce foreign genes derived from different organisms by Agrobacterium and particle bombardment techniques have enabled aggressive genome manipulation. Many genetically modified (GM) transgenic crops harboring foreign genes with useful traits, such as herbicide-tolerant and insect-resistant traits, have been developed in the last twenty-five years, and a wide variety of foods derived from GM plants are eaten worldwide.

Risk assessments of foods derived from transgenic organisms had been discussed, and the Codex Alimentarius Commission (CAC) authorized the principles and guidelines for assessing food safety derived from recombinant-DNA plants, animals, and microorganisms^1^^,^^2^^,^^3^^,^^4^^)^. Following these Codex guidelines, safety assessment procedures for seed plants and microorganisms harboring recombinant DNA were established by the Food Safety Commission of Japan^5^^,^^6^^)^. According to this procedure, the safety of >300 foods derived from GM seed plants have been authorized until 2020^7^^)^. From the perspective of risk regulation, the introduced foreign genes of transgenic organisms need to be detected using molecular biological techniques, which enable the identification of transgenic crops versus non-transgenic ones, even in some processed foods derived from transgenic crops and vegetables^8^^,^^9^^,^^10^^,^^11^^)^.

A novel molecular biological technology, genome editing using zinc-finger nucleases (ZFNs), and transcription activator-like effector nucleases (TALENs) and clustered regulatory interspaced short palindromic repeat (CRISPR)/CRISPR-associated 9 (Cas 9) RNA-guided DNA endonucleases (CRISPR/Cas 9), provides a novel approach for the next generation of plant breeding^12^^,^^13^^,^^14^^,^^15^^)^. Coupled with genome editing, oligo-directed mutagenesis, cisgenesis and transgenesis, RNA-directed DNA methylation (RdDM) and grafting using transgenic plants are called “New Plant Breeding Technology (NPBT)”^16^^,^^17^^)^. The remarkable property of genome editing and oligo-directed mutagenesis is that nucleotide sequences of genomic DNA are modified to create phenotypical traits providing benefits for producers and consumers, and such modification of genomic sequences is indistinguishable from those of naturally mutated varieties and artificial mutants caused by reagents and radiation. RdDM does not alter the nucleotide sequences of genomic DNA, but it does make the methylation status heritable and give rise to new phenotypes. Cisgenesis and transgenesis result in transgenic plants with the rearrangement or introduction of genomic DNA fragments derived from hybridizable species, whose alteration of nucleotide sequences is expected to be possible in natural hybridization or variants. In all the cases mentioned above, whole cells in their individual plant bodies are homogeneous with regard to their managed genomic and epigenomic status. However, grafting using transgenic and non-transgenic plants could enable the production of chimeras consisting of GM and non-GM plant parts in one plant body.

Grafting is a traditional technique^18^^,^^19^^)^. In general, wild plants that are the same species as cultivars, are more tolerant of abiotic and biotic stresses than cultivars. Therefore, cultivars, which are superior for producing food, are grafted as scions onto wild rootstocks. In some cases, the rootstock is prepared from a plant species different from the scion species^20^^,^^21^^,^^22^^)^. For example, most watermelon fruits are produced from the watermelon (*Citrullus lanatus*) scion grafted onto the bottle gourd (*Lagenaria siceraria*) rootstock. Undoubtedly, the rootstock and scion regions of these chimeric plant bodies are composed of different genomes. When the lower parts of GM plants are used as rootstocks and the upper parts of non-GM plants are used as scions, the fruit produced on the non-GM scion do not contain introduced DNA. Many grafted chimeric crops consisting of GM rootstocks and non-GM scions have been reported for fruits, such as grape^23^^)^, plum^24^^)^, blueberry^25^^)^, apple^26^^)^, cherry^27^^)^, pear^28^^)^, and citrus^29^^)^ plants, and vegetables, such as tomato^30^^,^^31^^,^^32^^)^, potato^33^^)^, cucurbits^34^^)^, pumpkin^35^^)^, eggplant^36^^)^, and soybean^37^^)^ plants. The transmission of useful traits from intraspecies grafting between GM rootstocks and non-GM scions has been reported, such as for non-GM watermelon onto GM bottle gourd^38^^)^ and non-GM potato onto GM-tomato or GM-tobacco^39^^)^. Furthermore, multiple grafting among tomato, bell pepper and eggplant^40^^)^ and among tobacco (*Nicotiana benthamiana*), tomato, and potato^41^^)^ of GM and non-GM scions and rootstocks have been reported.

Local short-range movement, especially for large molecules, occurs via plasmodesmata connection between neighboring cells. In addition, small chemical substances as well as RNAs and proteins can move from the top to the bottom of a plant body and vice versa, primarily through the vascular tissue and phloem (called long-range movement)^32^^,^^42^^,^^43^^,^^44^^,^^45^^,^^46^^,^^47^^,^^48^^,^^49^^)^. The latter movement occurs beyond the grafting adhesion from rootstock to scion and from scion to rootstock^20^^,^^50^^,^^51^^,^^52^^)^. For example, chimeric poplar with GM rootstock harboring the *cry* gene produced Bt toxin (Cry1Ac protein) derived from *Bacillus thuringiensis* and the non-GM scion showed resistance to leaf-eating insects in the scion parts of the plant, in which Bt toxin could be detected^53^^)^. Some peptides, such as flower-timing regulation^25^^,^^31^^,^^54^^,^^55^^)^, were moved through the junction of the scion and rootstock. In contrast, there is a report showing that large molecules of mRNAs and proteins were not transported from rootstocks to scions^38^^)^. When plant growth regulators are over-synthesized by the introduced genes in GM rootstocks, the phenotypic traits of non-GM scions are altered by the increase in plant growth regulators transmitted from rootstocks^56^^,^^57^^,^^58^^)^.

Most of these grafting experiments have not considered the food safety of the edible parts of plants, such as of fruits or tubers. However, the risk of edible plant parts to human health could be affected by the transport of toxic substances. The alkaloids of Solanaceae are synthesized in the roots and then transported to the rest of the plants. Recently, a severe food poisoning accident occurred in Japan when eggplant fruits from a grafted plant composed of angle trumpet, *Datura stramonium*, rootstock and eggplant, *Solanum melongena*, scion were cooked with pasta and eaten^59^^)^. Datura alkaloids were synthesized in the rootstock^60^^)^ and transported into the eggplant fruits in the scion. In grafted plants with tobacco rootstock and tomato scion, nicotine synthesized in the rootstocks was transported and accumulated in the leaves of the tomato scions^61^^)^. These examples highlight some of the risks that should be considered regarding the edible parts of scions in grafted plants.

Tomatoes are one of the major fruit crops using grafted seedlings prepared by farmers and nursery companies to provide virus- and pathogen-resistant plants^62^^,^^63^^,^^64^^)^. The characteristics of grafted tomato plants have been examined from the perspective of scientific interest^30^^,^^31^^,^^32^^,^^43^^,^^50^^,^^65^^,^^66^^)^ but not from the perspective of the food safety of tomato fruits. As a foundation of the assessment of the food safety of grafted plants, we conducted a multi-omics analysis of tomato fruits obtained from the grafted plants with a GM-tomato rootstock. A model tomato variety, “Micro-Tom” was transformed using a transgene encoding β-glucuronidase (GUS) driven by cauliflower mosaic virus 35S promoter (CaMV35S), and it was used as a rootstock. A non-GM scion was prepared from a commercial variety of mini tomato, “Stella Mini”. The GUS protein has been used as a versatile reporter of gene expression in plant molecular biology, and it is considered to be independent of plant metabolism^67^^)^. After grafting, the tomato fruit on the scion was analyzed. We examined the alteration of transcriptomic, proteomic, and metabolomic traits with food ingredients in fruits to contribute to the assessment of the food safety of grafted GM plants.

## 2. Materials and Methods

### 2.1 Preparation of Rootstock Plants of CaMV35S-*GUS* Introduced Micro-Tom and Scion Plants of Cultivar Tomato

The non-transgenic Micro-Tom (N-MT) and transgenic Micro-Tom (T-MT) plants that express the *GUS* gene under controlled CaMV35S promoter were kindly provided by Dr. Satoko Nonaka from the University of Tsukuba, Japan. The T-MT was generated by Agrobacterium-mediated transformation with pIG121-Hm^68^^)^. The Stella Mini Tomato (*Solanum lycopersicum* var. cerasiforme) is a true-bred cherry tomato cultivar and was brought from Noguchi Seed, Saitama, Japan. T-MT, N-MT, and Stella tomatoes were cultured for five weeks in a culture room, at 25°C with a light/dark cycle of 16 h of light provided by fluorescent light and 8 h of dark, and they were used as donors for the grafted plants. Stella Mini Tomato plantlets were used as the scions of the grafted plants, and they were grafted on T-MT, N-MT, and Stella rootstocks. We generated three different grafted plants consisting of Stella scion and T-MT rootstock (ST1, 2 and 3) and three consisting of Stella scion and N-MT rootstock (SN1, 2 and 3). After habituation in the culture room, grafted tomatoes were transferred into 15-cm diameter pots in a screened greenhouse. The fruit development statuses of the plants were determined based on the breaker stage, which is the stage when change in pericarp color is observed. Fruits were harvested at 5 and 10 days after breaker (DAB) and chilled quickly using liquid nitrogen. Then, they were stored at −80°C until use for analysis. Three fruits from each of the six grafted plants were independently subjected to analyses to provide biological triplicates.

### 2.2 Qualitative Genomic PCR

The leaves collected from T-NT and N-MT at 5 week-after-sowing and genomic DNAs were prepared as templates of PCR by the method described by Thomsom and Henry^69^^)^. The primer pairs to detect *GUS*^70^^)^ and *neomycin phosphotransferase II* (*NPTII*)^71^^)^ genes and the sequence of CaMV35S^72^^)^ for introduced transgenes and tomato endogenous *polygalacturonase* (*PG*) gene^73^^)^ were prepared according to previous reports (**Supplementary Table S1**). The reaction volume of 20 μL contained 10 μL of AmpliTaq Gold 360 (Applied Biosystems, Thermo Fisher Scientific, Waltham, MA, USA), 0.3 μmol/L each of primer pair, and an aliquot of the template. The PCR reaction was performed with TP600 (Takara Bio Inc., Shiga, Japan) according to the following step-cycle program; per-incubation 95°C for 5 min, following by 35 cycles of denaturing at 95°C for 15 s, annealing at 55°C for 15 s, and extension at 20 s in each cycle. The PCR products were separated by 2% agarose-TBE gel.

### 2.3 Transcriptome Analysis of Fruits Prepared from Grafted Tomatoes of Rootstock Plants of CaMV35S-*GUS* Introduced MT and Scion Plants of Cultivar Tomato

#### 2.3.1 Total RNA Extraction and RNA-seq Data Analysis

Total RNA from tomato fruits stored at −80°C was extracted using the FavoPrep Plant Total RNA Mini Kit (Favogen Biotech Co., Ping-Tung, Taiwan) and by following the instructions provided. The outsourcing service of Eurofins Genomics (Tokyo, Japan) constructed the preparation of RNA library and obtained the mRNA sequencing data. The mRNA purified by poly(A)^+^ and paired-end 101-base sequencing data was generated using HiSeq 4000 (Illumina Inc., San Diego, CA, USA). The mRNA-seq data (BioProject ID: PRJDB9192) were obtained with a total of 48.9 million reads. Then, 46.4 million reads were obtained after trimming the reads containing adapter sequences, poly-N, low-quality, and discorded fragments less than 50 bp using the quality control tool, fastp (v0.20.1). After the fastp data were mapped against the tomato transcriptome data ITGA4.0^73^^)^, the gene expression levels were calculated using Salmon (v0.14.1). The NumReads estimate, Salmon’s estimate of the number of reads mapping to each transcript, was normalized by a trimmed mean of M values (TMM) using the R package (v3.6.1), edgeR (v3.28.1). Hierarchical cluster analysis was performed using the ward.D2 method in the R package stats (v3.6.1).

#### 2.3.2 Identification of Differentially Expressed Genes (DEGs)

DEGs were identified between ST and SN using the edgeR package on TMMs. A DEG was declared if an associated false discovery rate p-value (P_FDR_) < 0.05 was observed. Gene expression levels of DEGs were used to generate a heatmap using two R packages: heatplus (v2.32.0) and genefilter (v1.68.0).

#### 2.3.3 Blast Search on Transcriptome Assembly

ST mRNA-seq data were concatenated in two files and inputted into the assembler Trinity (v2.8.6) with standard parameters to investigate the transferring *GUS* gene or movement of *GUS* gene transcripts from the rootstock to fruits of the scion. The tblastn (blast+ v2.9.0) search in the generated transcriptome data was used against the *GUS* transgene (AAC53703).

### 2.4 Proteome Analysis to Tomato Fruits

#### 2.4.1 Preparation of Digested Protein Samples

A measure of 100 mg of pulverized fruit materials was suspended in 500 μL of CellLytic P extraction buffer (Sigma-Aldrich Japan Co., Tokyo, Japan). The resulting suspension was vortexed for 1 min and then centrifuged at 10,000 rpm for 10 min. The insoluble materials were removed using filtration through a membrane filter (0.45 μm; Merck Millipore, Billerica, MA, USA). Then, 100 μL of 5 mM iodoacetamide was added to the resulting filtrate and incubated in the dark for 10 min, followed by centrifugation for 30 min. Next, trypsin was added and incubated overnight at 37°C. After filtration through the membrane filter, the filtrate was acidified with trifluoroacetic acid and stored at −80°C until analysis.

#### 2.4.2 Ultra-High Performance Liquid Chromatography (UHPLC)-Mass Spectrometry Analysis and Multivariate Analysis

The essential protocols for UHPLC and data analysis were conducted following a previous report^74^^)^. The UHPLC system was interfaced with a Q Exactive hybrid quadrupole-orbitrap mass spectrometer (Thermo Fisher Scientific). A 2 μL portion of each sample was introduced using full-loop injection into an UltiMate 3000 RS LC system with a photodiode array detector (Thermo Fisher Scientific). Separation was performed using an Acquity UHPLC BEH-C18 column (1.8 μm, i.d. 2.1 × 100 mm; Waters Co., Milford, MA, USA) at 40°C. The mobile phase consisted of a 0.1% aqueous solution of formic acid (phase A) and acetonitrile containing 0.1% formic acid (phase B) running at a flow rate of 0.1 mL/min. The parameter of gradient elution was 5%–40% B in the initial 15 min, 40%–95% B in successive 75 min increments, holding for 15 min, and then returned to 5% B in 0.2 min. Mass spectrometry (MS) was measured in the positive- and negative-ion electrospray modes. Nitrogen was used as the desolvation gas at 300°C. The capillary and cone voltages were set to 4,000 V and 35 V, respectively. Data were collected over the range of *m/z* 150–2000 and were centroided during the acquisition. MS/MS data were acquired in DDA mode, using the Top20 method. The MS1 mass range was 133–2000 *m/z*, and the resolution was set to 60000 (at 400 *m/z*), the AGC target was 1e6 and maximum injection time was set to 120 msec. The MS/MS resolution was set to 17500, with an isolation window of 2 *m/z*, underfill ratio of 1.3%, AGC target of 5e5, and maximum injection time of 100 msec. Dynamic exclusion was set to 120 msec.

All data obtained from the four assays in the two systems in both the positive- and negative-ion modes were processed using Progenesis QI data analysis software (Nonlinear Dynamics, Newcastle upon Tyne, UK). This was used for peak picking, alignment, and normalization to produce peak intensities for retention time and *m/z* data pairs. The ranges of the automatic peak picking assays were between 5 and 100 min. The resultant data matrices were imported into SIMCA version 14.0 (Umetrics, Umeå, Sweden) for further multivariate statistical analysis with Pareto scaling.

### 2.5 Metabolome Analysis of Tomato Fruits

#### 2.5.1 Liquid Chromatography-Electrospray Ionization-Mass Spectrometry Analysis

Metabolites were extracted following Iijima et al^75^^)^ with some modifications. Frozen tomato fruits were lyophilized and ground into powder in liquid nitrogen. A measure of 30 mg of ground sample was mixed with 900 µL of 75% methanol containing reserpine (20 µg/mL) as an internal control. After homogenization using a Mixer Mill MM 400 (Retsch, Haan, Germany) with a zirconia bead at 30 Hz for 2 min, the homogenate was centrifuged at 12,000 × *g* for 10 min at 4°C. The extraction was repeated twice, and the supernatants were combined in a new microcentrifuge tube. The supernatant was filtered through a PTFE membrane (0.2 µm; Millex-LG; Merck Millipore).

Non-targeted metabolite analysis was conducted using liquid chromatography-electrospray ionization-MS (LC–ESI-MS) in an LCMS-8040 system with control from LabSolutions software (Shimadzu Corp., Kyoto, Japan). A measure of 5 µL of filtrated sample was separated on the Kinetex C18 column (2.6 μm, i.d. 2.1 × 150 mm; Phenomenex Inc., Torrance, CA, USA) at 40°C with a flow rate of 0.3 mL min^−1^. An initial solvent of 5% acetonitrile/0.1% formic acid (v/v) was applied for 2 min. Then, an acetonitrile concentration gradient (5%–98%) was applied in the presence of 0.1% formic acid over 7 min, followed by a 3-min elution with 98% acetonitrile/0.1% formic acid. Mass spectra within the *m*/*z* range of 100–1500 were obtained using Q3 scan mode with positive/negative polarity switching. The MS conditions were a DL temperature of 250°C, nebulizer gas flow rate of 3.0 dm^3^ min^−1^, heat block temperature of 400°C, and drying gas flow rate of 15 dm^3^ min^−1^. A dataset of LC-ESI-MS raw data files was converted to mzXML file format using ProteoWizard’s MSConvertGUI software^76^^)^, and the mzXML files were uploaded to XCMS Online ver. 3.7.0^77^^)^ to process the dataset. The mass data obtained between 2 and 12 min were analyzed using the XCMS and a provided parameter set #11025 with the feature detection method “matchFilter”. α-Tomatine was identified using an authentic standard compound.

#### 2.5.2 Gas Chromatography-Electron Ionization-Mass Spectrometry Analysis

Metabolite extraction, derivatization, and gas chromatography-electron ionization-MS (GC-EI-MS) analysis of tomato fruit samples were conducted at the Laboratory of Biomolecule Analysis, Kazusa DNA Research Institute (Kisarazu, Chiba, Japan).

Metabolites from 10 mg of lyophilized tomato fruit were extracted using 1 mL of 80% methanol containing 10 µg of ribitol as an internal standard, and tissue debris was removed by centrifugation at 15,000 rpm for 5 min. The supernatant was passed through a Monospin C18 column (GL Sciences Inc., Tokyo, Japan), and a 20 µL sample was dried under a nitrogen gas stream. The dried sample was derivatized for 90 min at 30°C in 50 μL of 20 mg/mL methoxyamine hydrochloride in pyridine followed by a 30-min treatment at 37°C with 50 μL of *N*-methyl-*N*-(trimethylsilyl)trifluoroacetamide.

GC-MS analysis was performed on a SHIMADZU QP-2010 Ultra system (Shimadzu) equipped with an Agilent DB-5 column, 30 m × 0.25-mm inner diameter with a 1.00-µm film thickness (Agilent Technologies Inc., Santa Clara, CA, USA) and injection volume was 0.5 µL. The injection port temperature was 280°C. The helium gas flow rate through the column was 1.1 mL min^−1^. The oven temperature was initially kept at 100°C for 4 min, and it was increased from 100°C to 320°C at 4°C/min and then kept at 320°C for 8 min. The transfer line and ion-source temperatures were 280°C and 200°C, respectively. Mass spectra generated at an ionization energy of 70 eV were acquired from 45 to 600 *m*/*z* with a scanning frequency of 2,000 u/sec.

The AnalyzerPro (SpectralWorks, Runcorn, UK) and FragmentAlign programs^78^^)^ were used for spectral data mining for non-targeted analysis. GCMSsolution program (Shimadzu) was used with GC/MS Metabolite Database Ver.2 (Shimadzu) for metabolite annotation.

#### 2.5.3 Statistical Analysis

After creating the multivariate data matrix, principal component analysis (PCA) and volcano plot analysis were performed with the web-based free software MetaboAnalyst 4.0^79^^)^. The data scaling used for PCA was auto scaling, which is mean-centered and divided by the standard deviation of each variable. For the volcano plot analysis, the fold change threshold was set at 2.0, and the P_FDR_ threshold was set at 0.05.

### 2.6 General Food Ingredient Analysis

Analyses of the water, protein, lipid, ash, and carbohydrate contents of the tomato fruits were conducted by the Japan Food Research Laboratories (Tokyo, Japan). Triplicate samples of 30 g of frozen tomato fruits, each of which was approximately 10 fruits harvested from each plant line, on 7 DAB were ground using a food mixer (IFM-C20G; Iwatani Co., Osaka, Japan) and then sent to the Japan Food Research Laboratories. The data were statistically analyzed using Tukey’s honestly significant difference (HSD) tests in R software (version 4.0.3 (2020-10-10)) (The R Foundation for Statistical Computing, Vienna, Austria).

## 3. Results and Discussion

### 3.1 Properties of Traits of Plants and Fruits of Stella Mini Tomato Scions Grafted on T-MT (ST) and on N-MT (SN)

**Fig. 1.**
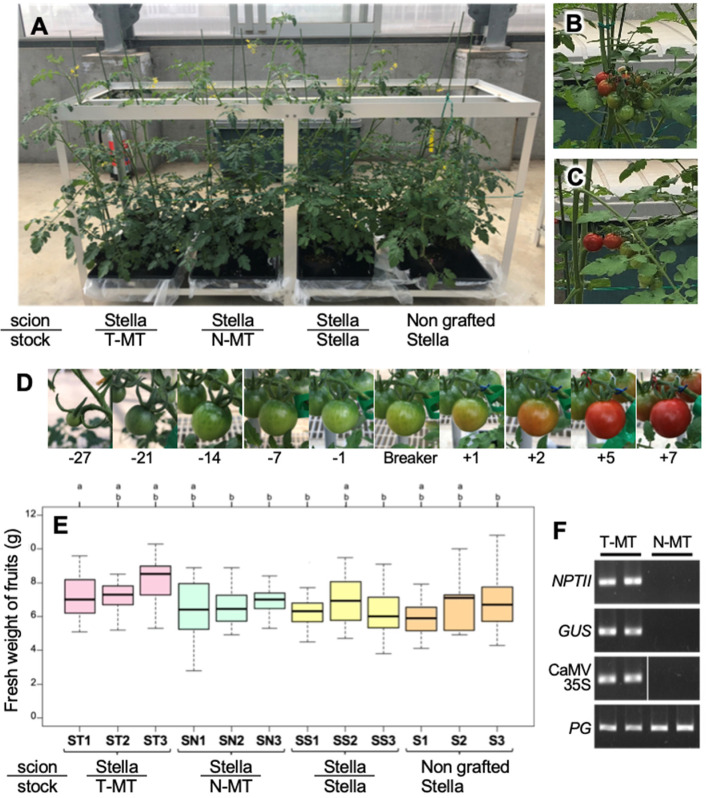
Phenotype of non-transgenic Stella Mini Tomato (S) scions grafted on transgenic and non-transgenic Micro-Tom (T-MT and N-MT, respectively) as rootstocks. (A) Aerial parts of plant shapes of S scions grafted on respective T-MT and N-MT at the beginning of the flowering stage (mid of June in 2019). Stella scions grafted on Stella rootstocks and non-grafted Stella are shown as the control. (B, C) Phenotypes of ripe fruits settled on S scions grafted on respective T-MT (B) and N-MT (C). (D) Fruit ripening stages settled on non-grafted Stella. Fruits past 10 DAB of 7 DAB defined the fully ripened stage. (E) Fresh weight of fruits settled on Stella scions grafted on T-MT, N-MT, and Stella rootstock and non-grafted Stella harvested at 7 DAB. Different letters above the box plots indicate significant differences among the grafted plants according to the Tukey-HSD test (ɑ = 0.05). (F) Detection of transgenes for *NPTII* and *GUS* genes and CaMV 35S promoter region in the T-MT. As PCR control, a primer pair for the tomato endogenous *PG* gene was used. No amplified products for transgenes could be detectable in N-MT.

T-MT, N-MT and Stella Mini Tomato were cultured for five weeks in the culture room and provided as donors for grafted plants. MT is a dwarf variety of tomato plant that is regarded as an experimental model plant for genomics and molecular breeding studies of tomato plants^80^^)^. The Stella Mini Tomato, *Solanum lycopersicum* var. cerasiforme, is a true-bred cherry tomato cultivar, and it was used to represent an edible cultivar in this study. The grafted plantlets were transferred to the screened greenhouse in the middle of April 2019. All tomato plants including the combinations of grafted and non-grafted plants, flowered until the middle of June 2019 (**Fig. 1A**). The flowers were tapped gently to promote pollination as soon as they bloomed. The successfully-pollinated flowers settled the breaker stage fruit approximately one month after flowering. The presence of the transgene in T-MT was detected using PCR for the *NPTII*, *GUS* gene and CaMV 35S promoter sequences (**Fig. 1F**). In the early growth stage, Stella scions grafted on MT rootstocks were smaller than those grafted on Stella rootstock, but no differences were observed in the aerial parts of the plant shapes (**Fig. 1A–C**). Stella scions grafted on MT rootstocks were slightly faster at flowering than those grafted on Stella rootstocks (data not shown). Fruits of the non-grafted MTs and the grafted plants with MTs as scions were fully ripe at 5 DAB, whereas fruits of the non-grafted Stella and grafted plants with Stella scions did not appear to be fully ripe. However, the cleavages were observed in many fruits at 10 DAB. Thus, 7 DAB was defined as the fully ripe stage of the Stella plants (**Fig. 1D**), and was used for the following analyses. The grafted plants with Stella scion and MT rootstock did not differ in their fruit development rates and fresh weight compared to the self-grafted Stella and non-grafted Stella (**Fig. 1E**). Additionally, among the grafted plants with Stella scion and N-MT or T-MT rootstock, there were no differences in the fruit development rate and fresh weight regardless of the presence or absence of transgenes of MT rootstock (**Fig. 1E**). These results suggest that grafting operations on MT rootstocks did not affect fruit formation in Stella scions. In addition, it was suggested that the expression of the *GUS* gene in MT rootstock did not affect the morphology, growth, and fruit formation of the Stella scion of grafted plants.

### 3.2 Transcriptome Analysis in Tomato Fruits

#### 3.2.1 Characterization of Transcriptome Data on Stella Scions grafted onto T-MT and N-MT rootstocks

**Fig. 2.**
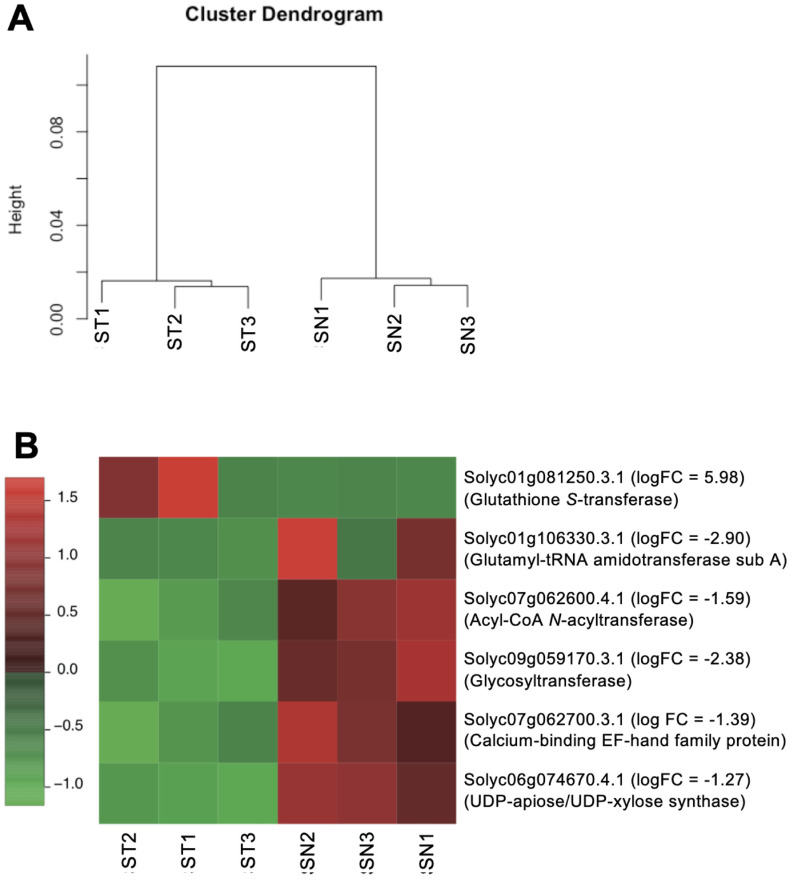
(A) Hierarchical cluster tree of transcripts in fruits derived from ST1, 2, and 3 lines and SN1, 2, and 3 lines. Dendrogram generated from 22,035 genes expressed at least in one of the six samples. (B) Analysis of expression patterns of DEGs. Functional annotation of each DEG and logFC (ST vs. SN) are shown for the ST and SN fruits.

We used grafted tomato plants comprising of non-transgenic Stella tomato as the scion and a transgenic MT rootstock expressing the *GUS* gene under the control of the CaMV35S promoter (ST line). In addition to these grafted tomato plants, we analyzed SN lines consisting of a non-GM Stella scion and non-transgenic MT rootstock. The 46.4 million RNA-seq reads from the fruits harvested from three lines of ST1, 2 and 3 and SN1, 2 and 3 against the ITGA4.0 reference transcriptome data, including 33,976 genes, showed that 84.5% reads were mapped in total. Overall, the mapped reads enabled the identification of a total of 22,035 genes expressed with NumReads > 0, at least in one of the six samples. The total number of expressed genes of ST was 18,864 at ST1, 18,738 at ST2, 18,643 at ST3, and of SN were 18,306 at SN1, 18,961 at SN2, 18,374 at SN3. The hierarchical cluster analysis result indicated that the expression profiles are similar in ST and SN, respectively (**Fig. 2A**). Thus, we investigated DEGs between ST and SN.

#### 3.2.2 Identification of Differentially Expressed Genes

The transcriptomes of three biological replicates of ST and SN were compared and DEGs were identified. This allowed the identification of six DEGs (P_FDR_ < 0.05), and only one gene was more expressed in ST than SN, and the others were downregulated in ST (**Fig. 2B**). The DEGs functional annotation showed that the highly expressed gene in ST is glutathione *S*-transferase, and the other five less expressed genes in ST were involved in some of the transferases. However, β-fructofuranosidase, polygalacturonase 2A, pectinesterase, and superoxide dismutase are known as tomato allergen^81^^)^, and they were not detected as DEGs in the comparison between ST and SN.

#### 3.2.3 Investigation of the Transfer of the GUS Gene from Rootstocks to Scions

The ST mRNA-seq data were assembled to generate a transcriptome data, and the resulted profile of the transcriptome data is as follows: the number of contigs 53,530, N50 length 1,880, maximum contig length 15,831. We searched the *GUS* gene via BLAST search with this transcriptome dataset. We did not detect any transcripts derived from the *GUS* gene in the ST transcriptome data. It is possible that the *GUS* RNA fragments exist at a lower level in the ST and we fail to detect these fragments in the ST mRNA-seq data. Even so, such small amounts of the *GUS* RNA fragment had a limited effect on the transcriptome in the ST.

### 3.3 Proteome Analysis in Tomato Fruits

We designed a strategy to comprehensively extract fragment peaks from UHPLC-MS chromatograms of digested mixtures of proteins from tomatoes and to compare these between the SN and ST groups. The data matrices were prepared using peaks obtained from peak extraction on the chromatograms of tomato samples (6 samples, ***n*** = 5) measured using UHPLC-MS.

**Fig. 3.**
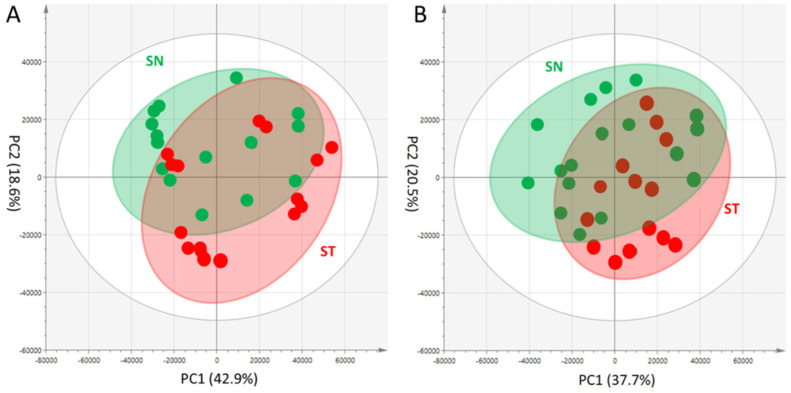
Comparison of digested protein composition in fruits from grafted tomato plants. (A) PCA score plot of peptide analysis by UHPLC-ESI-MS obtained in positive mode. (B) PCA score plot of peptide analysis by UHPLC-ESI-MS obtained in and negative mode. Each plot represents an individual analytical sample. Percentage values in parentheses are the respective contribution ratios.

In the UHPLC–ESI-MS analysis, we detected 1,487 and 955 peaks from peak extraction from the chromatograms of positive-ion mode and negative-ion mode, respectively. A PCA of a total of 30 samples was performed using data matrices representing peak intensities extracted from individual peak groups^82^^)^, and we compared the extracts primarily includes digested peptide fragment contents between ST and SN lines. A PCA with high statistical values of *Rx*^2 ^(0.847) and *Q*^2^ (0.702) was derived from the UHPLC-ESI-(+)-MS, where, *Rx*^2^ represents the goodness of fit and *Q*^2^ reveals the predictability of the PCA model. The projection of PCA models using projections into two dimensions of the first principal component (PC1) and the second principal component (PC2) is shown in Fig. 3A. The two groups seemed to form their own clusters, but the two groups largely overlapped (Fig. 3A). Therefore, no significant difference between the two groups of SN and ST was observed. Subsequently, the PCA derived from UHPLC-ESI-(-)-MS was examined. High statistical values for *Rx*^2^ (0.883) and *Q*^2^ (0.745) were obtained from the PCA. Fig. 3B presents the projection of the PCA models in PC1 and PC2. In this PCA score plot of UHPLC-ESI-(-)-MS, no significant difference was observed between the two groups of SN and ST, which is similar to the results for UHPLC-ESI-(+)-MS. Also, no obviously different peptide fragments were identified as contributing components.

From this fingerprinting of tomato fruits by UHPLC-ESI-MS, we found that *GUS* gene expression in rootstock had a limited effect on the production of proteins in tomato fruits. Recently, there were reports on proteomic analysis using Solanaceae plants for the study of the change of membrane proteins during ripening^83^^)^. Our analysis using UHPLC-ESI-MS revealed that the contents of crude protein extracts were not different between the ST and SN fruits. Further analytical and statistical studies are needed to confirm differences between the recombinant and non-recombinant forms, and further studies are currently underway.

### 3.4 Metabolome Analysis in Tomato Fruits

#### 3.4.1 Analytical Scheme

We have explored the possibility of unanticipated metabolomic changes was exerted in non-transgenic scions of grafted crop plants engrafted with rootstocks carrying transgenic events. Metabolomic profiles were obtained using analytical data from non-targeted metabolomic profiling by LC-ESI-MS and GC-EI-MS. In mass chromatograms, metabolites are separately eluted and detected as peaks of a set of ion signals within certain time ranges. Such peaks comprised of ions generated from a single metabolite, exhibiting *m*/*z* values of molecular ions, fragment ions, and isotopic ions, are compiled into peak groups representing molecular information from individual metabolites. Thus, in this study, each peak group should represent a distinct metabolite.

#### 3.4.2 Metabolomic Profiling of Tomato Fruits from Grafted Plants

Fruits of 10 DAB stage were harvested from these grafted plants and subjected to LC-ESI-MS and GC-EI-MS analyses. Three fruits harvested from each independent plant line of ST1, 2, and 3 and from each line of SN1, 2, and 3, making 18 fruits in total, were used for the MS analyses.

**Fig. 4.**
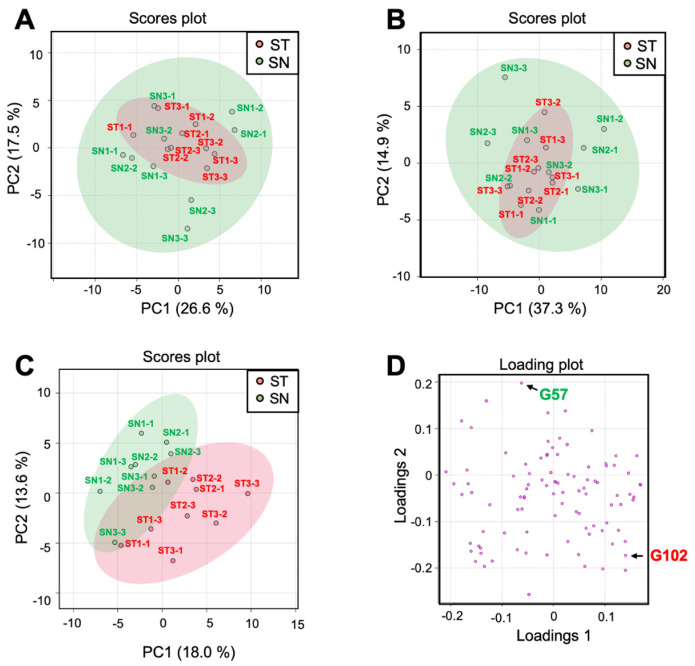
Comparison of metabolite composition in fruits from grafted tomato plants. (A, B) PCA score plots of metabolite analysis using LC-ESI-MS obtained in positive mode and negative mode, respectively. Each plot represents an individual analytical sample. Percentage values in parentheses are the respective contribution ratios. (C) Comparison of metabolite compositions in fruits from the grafted tomato plants using GC-EI-MS analysis. A PCA score plot of metabolite profile. Each plot represents an individual analytical sample. (D) Each plot represents an individual ion selected from each peak group. The numbers next to the plots represent their peak group ID. Tomato grafted plants, ST1, ST2, and ST3, were generated by grafting non-transgenic scions from Stella Mini Tomato onto transgenic Micro-Tom (MT) rootstocks expressing a *GUS* gene under the control of CaMV35S promoter. The control grafted plants, SN1, SN2 and SN3, were comprised of non-transgenic Stella scions and non-transgenic MT rootstocks. Three individual fruit samples from each grafted plant were subjected to MS analyses. Thus, three analytical results are shown for individual grafted plants (SN1-1, SN1-2, and SN1-3 from SN1 grafted plant, for example).

In the LC-ESI-MS analysis, we identified 60 and 33 peak groups in the analyses of positive ion mode and negative ion mode, respectively. Using representative peak intensities from individual peak groups, PCA was performed to compare the metabolite composition between the ST and SN lines. The PCA score plots composed of the first two PCs did not show metabolome cluster separation between ST and SN (Fig. 4A and 4B). Similarly, the PCA score plots of PC3 and PC4 did not show metabolome cluster separation between ST and SN (data not shown). The cumulative variances of first four PCs are 66.3% in the positive ion mode analysis and 70.0% in the negative ion mode analysis. There were not more than two-fold differences in the metabolite accumulation levels between ST and SN. In the GC-EI-MS analysis, we obtained 114 peak groups. Among them, 97 peak groups were subjected to PCA because the remaining 17 peak groups were not consistently detected in the analyzed samples due to their low abundance. Two-dimensional PCA score plot graph composed of the first two PCs showed overlapping but partially separated metabolome clusters derived from the ST and SN lines (Fig. 4C). On the other hand, the PCA score plots of PC3 and PC4 did not show metabolome cluster separation between ST and SN (data not shown). The cumulative variance of first four PCs is 49.9%. Two peak groups, G57 and G102, showed more than a two-fold difference in their relative abundance (P_FDR_ < 0.05) (Fig. 4D). G57 was annotated as asparagine, and its accumulation was 2.8-fold higher in the SN lines. The accumulation level of G102, whose identity and molecular structure are unknown, was three-fold higher in the ST lines. However, G57 and G102 were not detected in a non-negligible number of fruits. Therefore, it is necessary to examine in detail the data to obtain a better understanding (Supplementary Table S2). Asparagine (peak group G57) was detected in all the samples from the SN lines at a similar intensity, while it was detected in only six fruits from the ST lines: two out of three fruits from each line. The contents of the metabolite represented by peak group G102 was very low. Thus, G102 was not consistently detected among all the fruit samples. For example, it was undetected in the SN1 fruits and detected in two of three fruits from SN2.

From the metabolite profiling of tomato fruits using LC-ESI-MS and GC-EI-MS, we found that *GUS* gene expression in rootstock had a limited effect on tomato fruit metabolites. However, the molecular identity of G102 remains unknown because of its extremely low concentration and a lack of practical information to investigate its molecular structure, such as an MS spectral database and authentic compounds. Nonetheless, it was also detected in the measurements in the fruits ripened on the non-grafted Stella plants, and there is no information to evaluate its biological effects on the edible part of the plants. It remains a point in question whether such a minor component can be critical.

**Fig. 5.**
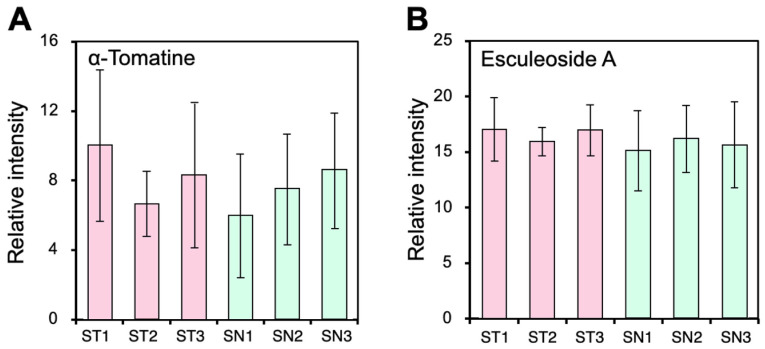
Contents of α-tomatine and esculeoside A in the ST and SN fruits. Relative signal intensity of peak group annotated as α-tomatine (A) and peak group predicted as esculeoside A (B). Error bars indicate standard deviation from three independent fruits from same grafted plant lines.

It is well known that Solanaceae plants contain toxic substances: steroidal glycoalkaloids^84^^)^. Representative steroidal glycoalkaloids in tomato fruits are α-tomatine and esculeoside A^85^^)^. In this study, we analyzed three independent fruits from each plant to compare these steroidal alkaloids using LC-ESI-MS. We found that the contents of α-tomatine and esculeoside A were not different between ST and SN fruits (Fig. 5A and 5B).

### 3.5 General Food Ingredient Analysis

**Fig. 6.**
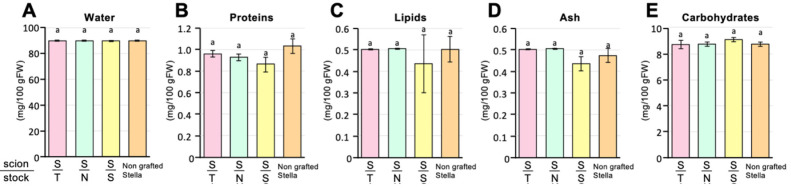
General food ingredients of fully ripened tomato fruits. The water, protein, lipid, ash, and carbohydrate contents of fruits settled on Stella scions grafted on T-MT and N-MT rootstocks (S/T and S/N, respectively) and Stella rootstock (S/S) and non-grafted Stella are displayed in A–E, respectively. Different letters at the above of the bars indicate significant differences among the grafted plants according to the Tukey-HSD test (ɑ = 0.05). Error bars represent standard error.

The contents of general food ingredients are shown in **Fig. 6**. No significant differences were found in the chemical contents of the fruits from grafted plants and a non-grafting Stella tomatoes (Tukey-HSD, ɑ = 0.05, ***n*** = 3) (**Fig. 6**). These measurements were closely related to the values described in the Standards Tables of Food Composition in Japan^86^^)^. These results suggest that the food ingredients of tomatoes with a Stella scion were not affected by either grafting operations or the type of the rootstocks used, which were T-MT, N-MT, and Stella.

## 4. Conclusions

We evaluated the effects of a *GUS* transgene in the rootstock on the grafted tomato fruits as a model case, since GUS is independent from the plant metabolism. The omics analyses revealed insignificant effects of grafting on the transcript, protein, and metabolite profiles of tomato fruits on the scion. In conclusion, grafting onto the GM rootstocks harboring the transgenes encoding the enzymes that do not affect the host metabolism had a quite limited effects on plant metabolism in the non-GM scion, indicating no apparent risk for food safety of the grafting onto the GM rootstock. The multiple omics analyses shown here could be applied for safety evaluation of the products of grafted plants in which the transgene products interact with the host metabolism. In contrast, grafted plants composed of GM scion onto non-GM rootstock will be developed especially in the cultivation of potato plants, and evaluation of food safety of the products from the non-GM rootstock would be required in future. Finally, the movement of the RNA molecules and translated protein product derived from the transgene through the grafted junction still remain uncertain and require further investigation in terms of food safety assessment.

## Supplementary materials

**Table:** Supplementary Table S1. Primer pairs for genomic PCR

Primer pair	Target	Amplicon length	Reference
5′-TTACGTCCTGTAGAAACCCC-3′	*GUS*	155 bp	Goda *et al.,* 2001^70^^)^
5′-TCGTTAAAACTGCCTGGCAC-3′			
5′-TGAATGAACTGCAGGACGAG-3′	*NPTII*	151 bp	Goda *et al.,* 2001^70^^)^
5′-AGGTGAGATGACAGGAGATC-3′			
5′-ATTGATGTGATATCTCCACTGACGT -3′	35S promoter	250 bp	Kuribara *et al.,* 2002^71^^)^
5′-CCTCTCCAAATGAAATGAACTTCCT -3′			
5′-GGATCCTTAGAAGCATCTAGT-3′	*PG*	384 bp	JRC Compendium of Reference Methods for GMO Analysis (QL-TAX-SL-001)^72^^)^
5′-CGTTGGTGCATCCCTGCATGG-3′			

**Table:** Supplementary Table S2. Relative intensity of G57 and G102 obtained using GC-EI-MS in the ST and SN fruits

Line	Fruit name	Relative intensity to internal control	
		G57	G102
SN1	SN1-1	0.087	n.d.*
	SN1-2	0.045	n.d.*
	SN1-3	0.095	n.d.*
SN2	SN2-1	0.063	0.033
	SN2-2	0.089	n.d.*
	SN2-3	0.105	0.071
SN3	SN3-1	0.116	0.035
	SN3-2	0.061	0.045
	SN3-3	0.058	0.078
ST1	ST1-1	0.035	0.095
	ST1-2	0.032	0.070
	ST1-3	n.d.*	0.119
ST2	ST2-1	0.048	0.106
	ST2-2	0.045	0.083
	ST2-3	n.d.*	0.106
ST3	ST3-1	n.d.*	0.076
	ST3-2	0.026	0.104
	ST3-3	0.050	0.104

* n.d, not detected. In statistical analysis shown in Fig. 4D, these missing values were replaced by 1/5 of the minimum positive values of their corresponding variables assuming that this was the detection limit.
